# Superior In Vivo Wound-Healing Activity of Biosynthesized Silver Nanoparticles with *Nepeta cataria* (Catnip) on Excision Wound Model in Rat

**DOI:** 10.1007/s12011-024-04268-4

**Published:** 2024-06-12

**Authors:** Berfin Rumeysa Sari, Sukriye Yesilot, Ozlem Ozmen, Cigdem Aydin Acar

**Affiliations:** 1 Department of Health and Biomedical Sciences, Burdur Mehmet Akif Ersoy University, Burdur, Turkey; 2 Department of Nursing, Bucak School of Health, Burdur Mehmet Akif Ersoy University, Burdur, Turkey; 3 Veterinary Faculty, Department of Pathology, Burdur Mehmet Akif Ersoy University, Burdur, Turkey

**Keywords:** Nanotechnology, *Nepeta cataria*, Silver nanoparticle, *In vivo*, Wound healing

## Abstract

Silver nanoparticles were biosynthesized with *Nepeta cataria* plant extract. It was determined that the synthesized Nc-AgNPs gave a strong absorbance peak at 438 nm wavelength in the UV-vis spectrophotometer. SEM and TEM analyses of Nc-AgNPs showed that the synthesized nanoparticles had a spherical morphology. Based on XRD analysis, the average crystallite size of Nc-AgNPs was calculated at 15.74 nm. At the same time, EDS spectrum analysis exhibited dominant emission energy at 3 keV, indicative of Nc-AgNPs. Nc-AgNPs showed an inhibition zone of 12 nm in gram-negative *Escherichia coli*, 10 nm in gram-positive *Enterococcus faecalis*, and 11 nm in *Staphylococcus aureus*. Nc-AgNPs showed high antioxidant properties, with 63% at 5000 μg/mL. The wound-healing properties of Nc-AgNPs were evaluated *in vivo* in wound models created in a total of 20 Wistar albino male rats, divided into four groups. After 10 days of treatment, the highest wound closure rate was seen in the Nc-AgNP + Vaseline (Group IV) treatment group, at 94%. It was observed that Nc-AgNP + Vaseline nanoformulation significantly increased wound healing, similar to Silverdin®, and Vaseline alone supported healing but did not result in complete closure. Histopathological examination revealed an increase in mature Type 1 collagen in Group IV and positive control (Group II), with better collagen maturation in vehicle control (Group III) compared to negative control (Group I). Immunohistochemical analysis showed complete epithelialization in Group IV and Group II, with distinct cytokeratin expressions, while Group III exhibited mild expressions.

## Introduction

Nanotechnology stands out as a highly promising technology of the twenty-first century, involving the practical application of nanoscience principles. It encompasses the observation, measurement, manipulation, assembly, regulation, and creation of materials at the nanoscale [1]. Nanomedicine is a new method of utilizing nanotechnological systems in illness detection and therapy. The discipline of nanotechnology is divided into two major categories: nanodevices and nanomaterials. Nanodevices are small devices that include microarrays and certain intelligent machines such as respirocytes. Nanomaterials have particles that are smaller than 100 nanometers (nm) in at least one dimension [2]. Nanotechnology-based solutions, particularly nanoparticles (NPs), have grown in popularity in medical research because they can provide considerable benefits in terms of efficacy and safety when compared to conventional therapeutic and diagnostic techniques [3, 4]. The properties of NPs vary based on their size and surface functions. Nanomaterials have a high surface-to-volume ratio due to their small size. Because of increased surface/volume ratios, it has more reactivity and thus effectiveness than bigger materials [5, 6]. Nanoparticles offer a wide range of therapeutic effects in medicine. For example, nanoparticles produced by green synthesis methods show strong cytotoxic and anticancer effects on cancer cells, making them promising agents in cancer treatment [7]. Thanks to their broad-spectrum antibacterial activities, nanoparticles are especially effective against pathogens such as antibiotic-resistant *Staphylococcus aureus* and *Escherichia coli* and accelerate wound healing. At the same time, integrating nanoparticles into wound dressings prevents microbial invasion, accelerates tissue regeneration, and reduces inflammation [8]. These findings highlight the potential for broad use of nanoparticles in medical applications. Some of the nanoparticles used in wound healing comprise silver, gold (Au), zinc (Zn), copper (Cu), nitrogen monoxide (NO), and antibiotics [9].

A wound is described as a disruption or damage to the normal anatomical structure and function [10]. Wounds pose a complex and dangerous threat to patients’ health and life [11]. Wounds can be caused by a variety of events, including surgical intervention, damage, and extrinsic factors such as pressure or shear, as well as underlying illnesses such as diabetes or vascular disease [12]. Wound repair is one of the most complex physiologic processes, requiring a plethora of distinct cell types, the contributions of which are strictly regulated over time [13]. Wound healing is a complex biological process that involves an intricate interplay of cellular processes such as inflammation, reepithelialization, and tissue restoration. All of these activities are regulated by several growth factors, cytokines, matrix proteins, and cell types [14, 15]. Wounds are classified as either acute or chronic. An acute wound is a skin injury that occurs suddenly. It could take 2–3 months to recover, depending on the depth and size of the skin in the epidermis or dermis layers. Chronic wounds, on the other hand, are more complicated wounds that heal more slowly. Chronic wounds are dangerous because they do not heal quickly [16, 17]. Wounds are sometimes called “silent epidemics” because if left untreated, they can lead to loss of limbs and even death [18]. Wound healing is a complex and carefully managed process that involves the regeneration, reformation, and repair of wounded tissues, all of which are critical for maintaining the skin’s barrier function. Acute wound healing is comprised of several sequential and overlapping phases, including hemostasis, inflammation, proliferation, and remodeling [18–20].

Non-healing wounds are a serious issue for both patients and healthcare systems. Bacterial colonization and biofilm formation are found in the majority of non-healing wounds. As a result, effective wound healing frequently necessitates antimicrobial therapy [21]. The twentieth-century discovery of antibiotics has revolutionized the fight against bacterial illnesses. Antibiotics are typically added to dressings to provide them with antibacterial activity [22]. However, drug resistance renders antibiotics and other antimicrobial treatments ineffective, and illnesses are becoming more difficult or impossible to treat [23]. The use of many antibiotics often leads to the development of multidrug resistance (MDR) against numerous harmful microorganisms. It is difficult to recover from infections that are resistant to several drugs, and doing so often necessitates several broad-spectrum antibiotic treatments that are harmful and expensive [24, 25]. Wound care presents difficulties for clinicians and researchers in a variety of medical domains. Silver nanoparticles (AgNPs) are considered promising prospects in this field due to their low cost and chemical stability, particularly their high efficiency and antibacterial activity against drug-resistant organisms in chronic wounds [26].

Due to its antibacterial effectiveness against a broad variety of microorganisms, silver nanoparticles have become noteworthy nanomaterials [27]. Silver has long been recognized to have antibacterial properties, and research indicates that it is safe for human usage in low quantities. While effective, some medicines have drawbacks such as toxicity and skin discoloration. According to studies, silver’s electrical structure is altered when particle size decreases, which enhances the metal’s antibacterial properties. This characteristic is linked to silver ions (Ag^+^)’s gradual oxidation and release in the biological environment. In addition to its ability to traverse the cellular membrane, Ag^+^ has an impact on cell division, which results in the microorganism’s demise. These unique qualities have increased interest in using silver nanoparticles in wound-healing studies [28, 29]. Silver nanoparticles exhibit antimicrobial properties, effective against various infectious organisms, including *Escherichia coli*, *Bacillus subtilis*, *Vibrio cholerae*, *Pseudomonas aeruginosa*, *Treponema pallidum*, and *Staphylococcus aureus* [30–32]. Many studies have been conducted on silver nanoparticles, and results from both *in vitro* and *in vivo* trials have demonstrated the antibacterial capabilities of AgNPs [11]. Silver nanoparticles have also demonstrated notable wound-healing properties. Research has indicated that silver nanoparticles can hasten wound healing and inhibit the production of scars in a dose-dependent manner. Research on animals has demonstrated the efficacy of medicinal products that incorporate silver nanoparticles in halting the production of pus in wounds, enhancing collagen alignment, and ultimately reducing the creation of scars [33]. *In vitro* research can provide some information on wound healing. However, tests on live animals demonstrate its wound-healing efficiency with more conclusive findings [34]. Adibhesami et al. reported that mice treated with AgNP had similar recovery times to gentamicin, an antibiotic used on the market to treat various types of bacterial infections [35].

The development of an oxidation reaction, in which silver ions (Ag^+^) are reduced by interaction with a reducing agent and converted into neutral atoms (Ag^0^), is the fundamental process leading to the synthesis of AgNPs. Different reducing agents can mediate this process via physical, chemical, or biological (green) methods [36]. There is a particular desire for green approaches that can create nanoparticles in an environmentally acceptable manner while maintaining long-term stability [37, 38]. Because physical or chemical techniques for the synthesis of metal nanoparticles have clear limitations and drawbacks, green approaches emerged as a new path in the chemical industry about two decades ago. AgNPs synthesized biologically have favorable characteristics including high yield, solubility, and stability, while AgNPs synthesized physically and chemically seem to need more effort and carry greater risk [39, 40]. The growing interest in environmentally friendly practices has led to the synthesis of AgNPs from a variety of sources, including plants, algae, bacteria, yeast, and fungi [41]. This has allowed for larger-scale manufacturing with lower levels of contamination [42]. Plant extracts are a promising source of biocompatible materials due to their abundance of bioactive chemicals. These chemicals can easily be extracted using water as a solvent, which is inert and environmentally friendly. Additionally, plant extracts can act as reducing and capping agents in the creation of nanoparticles. Recent research has demonstrated the potential of plant extracts as an alternative to conventional chemical synthesis methods, offering a safe and sustainable approach to nanomaterial fabrication [43, 44].

Wound dressings like gauze and cotton balls have limitations, such as easy adhesion to wounds and weak barrier function. Ideal wound dressings should prevent microbial invasion, reduce inflammation, accelerate wound closure, inhibit scar formation, and be cost-effective. These features are crucial for optimal wound-healing outcomes [45, 46]. Nanobiotechnology has been used to build medication, gene, and exosome delivery systems that can assist overcome these restrictions [47]. AgNPs enhance anti-inflammatory properties, have antibacterial action against a wide range of bacterial strains, can be included into wound dressings, and have surface changes that facilitate drug delivery, all of which help the wound-healing process [48].

The genus *Nepeta* belongs to the family Lamiaceae, which is rich in bioactive secondary metabolites. The word cataria derives from the Latin word “cathus,” meaning cat. Bioactive compounds of *Nepeta cataria* have been used since prehistoric times. *N. cataria* has been reported to have a wide range of biological activities, including analgesic, antiasthma, anticancer, anti-inflammatory and antimicrobial, antiarthritis, antipyretic, hepatoprotective, and antithrombotic [49–51].

In this study, *Nepeta cataria* plant extract was used and it was aimed at synthesizing silver nanoparticles with an environmentally friendly method, characterizing them, and examining their antibacterial and antioxidant effects. Additionally, the healing effects of silver nanoparticles integrated into pure Vaseline on the *in vivo* wound model created in rats were investigated, and it was aimed at examining the histopathological effect of silver nanoparticles on rats.

## Material and Methods

### Preparation of *Nepeta cataria* Extract

A dried *Nepeta cataria* plant was obtained from a local store for use in the biosynthesis process (Bioline, Lot No# 6970117121360). It was weighed (1 g) and dissolved in 100 mL of distilled water (dH_2_O). For 2 min, the mixture was boiled in a microwave oven (900 W). The plant extract was given time to cool down. The resulting plant extract was filtered using Whatman No. 1 filter paper after chilling, yielding *Nepeta cataria* aqueous plant extract [52].

### Silver Nanoparticle Green Synthesis and Ag-Vaseline Nanoformulation Fabrication

A 5 mM AgNO_3_ (silver nitrate) (Sigma Aldrich, St. Louis, Missouri, USA) solution was prepared with 100 mL of dH_2_O. *N. cataria* plant extract and AgNO_3_ solution were prepared in a ratio of 1:9 [53]. Ninety milliliters of a 5 mM AgNO_3_ solution and 10 mL of *Nepeta cataria* plant extract were mixed. The mixture was boiled in the microwave (900 W) for 2 min. Following the color change, the solution was centrifuged at 10,000 rpm for 15 min, the supernatant was discarded, and the process was repeated by washing with distilled water. Finally, washing with methanol was performed, and the Nc-AgNPs were dried at 40 °C overnight and ground into powder. To create Ag-Vaseline nanoformulation, powder Nc-AgNP was mixed in Vaseline at 1%.

### Characterization of Nc-AgNPs

The reduction of metal ions was visually examined in terms of the color change of AgNPs in the reaction medium. After visual examination, to characterize silver nanoparticles, UV-vis spectrophotometry, scanning electron microscopy (FE-SEM), energy-dispersive X-ray spectroscopy (EDS), and transmission electron microscopy (TEM) techniques were used. The UV spectrum of biosynthesized Nc-AgNPs was measured in the wavelength range of 300–600 nm using a UV-vis spectrophotometer (T60, PG Instruments Ltd., Japan). Determination of the surface morphology and size of Nc-AgNPs was carried out by FE-SEM and TEM analyses, and elemental analysis was carried out by EDS. X-ray diffraction (XRD) analysis was performed to characterize the crystal structures and phase identification of Nc-AgNPs.  The crystal domain size was calculated using *D*. Scherrer’s equation (*D* = *Kλ*/*β*cos*θ*) [54, 55], where *D* is crystallite size of AgNPs, *λ* is wavelength of the X-ray source (1.54056 Å), *β* is full width at half maximum (FWHM) of the diffraction peak in radians, *k* is the Scherrer constant that changes. 0.9 to 1, and *θ* is the Bragg angle in radians.

### Disk Diffusion Method

Antibacterial activity was determined by the Kirby-Bauer disk diffusion method against gram-negative *Escherichia coli*, gram-positive *Enterococcus faecalis*, and *Staphylococcus aureus* bacteria using Mueller-Hinton agar (MHA) with the measured inhibition zones in millimeters (mm). This method has been utilized in numerous investigations [56]. All of the bacterial strains were grown overnight at 37 °C in 10 mL of Mueller-Hinton Broth (MHB) in a 50-mL Erlenmeyer flask. The grown bacterial cell suspensions were then dispersed on MHA plates in 50-μL batches. In addition, 6-mm sterile disks saturated with positive control (penicillin/streptomycin), negative control (sterile water), and Nc-AgNPs (1000 μg/mL) were deposited on each plate and incubated for 24 h at 37 °C. After incubation, the diameter of the growth inhibition (mm) zones was measured using a digital caliper [57, 58].

### 1-Diphenyl-1-2-picrylhydrazyl (DPPH) Scavenging Assay

A solution of DPPH was prepared using methanol at a concentration of 0.1 mM. Nc-AgNPs were prepared at various concentrations ranging from 0 to 5000 μg/mL and mixed with the DPPH solution in a 1:1 ratio. The resulting mixture was kept in the dark at room temperature for 30 min. Spectrophotometric measurements were made on control test samples at 517 nm using a microplate reader. Ascorbic acid was used as a positive control. All measurements were taken three times. The radical scavenging activity was expressed as a percentage of inhibition [59].

$$\%\textrm{Inhibition}=\left(\textrm{Control}\hbox{--} \textrm{Sample}\right)/\textrm{Control}\ast 100$$

where % Inhibition is percentage of the radical scavenging activity; Control, absorbance of the control sample (DPPH solution without test sample); and Sample, absorbance of the test sample (DPPH solution with test compound).

### Experimental Design of Animals

This study involved 20 male Wistar albino rats who were 10 weeks old and weighed an average of 250 ± 50 g. The animals were obtained from the Experimental Animal Production and Research Center at Burdur Mehmet Akif Ersoy University. The rats were housed in a well-ventilated facility with a 12/12-h light/dark cycle and a constant temperature of 25 ±2 °C. The rats were fed standard rodent chow and tap water ad libitum. Before being employed in the study, the rats were given a week to adjust to their surroundings. The present study was approved by the Burdur Mehmet Akif Ersoy University Animal Experiments Local Ethics Committee with protocol number 30.03.2023/1025.

Twenty rats were randomly distributed into four groups of five rats each (Table 1). The groups were as follows: positive control group (1% silver sulfadiazine cream—Silverdin®), negative control group (no application), vehicle control group (vaseline), and nanoformulation (1% Nc-AgNP + Vaseline) group.

**Table 1:** Rats are divided into experimental and control groups

Experimental group	Number of animals	Implementation
I. Negative control	5	There are no applications
II. Positive control	5	1% silver sulfadiazine cream (Silverdin®)
III. Vehicle control	5	Vaseline
IV. Nanoformulation	5	Vaseline + Nc-AgNP (%1)

### Creation of Wound Model and Treatment

A rat excisional wound model was used to determine the effectiveness of Ag-Vaseline nanoformulation in wound healing. Before creating wounds, rats were weighed to establish the proper anesthetic dosage. For anesthesia, an intraperitoneal injection of a ketamine/xylazine combination (90–10 mg/kg) was administered. Following aseptic surgical techniques, two full-thickness excisional wounds of 5 mm circular diameter were created bilaterally on the dorsum of each rat using a biopsy punch [27].

Except for the negative control group, Silverdin^®^, Vaseline, and Nc-AgNPs + Vaseline were applied topically to the lesions every day, and lesion diameters were measured from the inner edges with a digital caliper (in mm) on days 1–4–7 and 10. Wound contraction was calculated as the percentage reduction in wound area as formulated below. Epithelialization time was recorded as the number of days required to ensure that no raw wound was left behind after the wound was opened [60].

$$\textrm{Wound}\ \textrm{contraction}\ \left(\%\right)=\frac{\left(\textrm{first}\ \textrm{day}\ \textrm{wound}\ \textrm{area}-\textrm{last}\ \textrm{day}\ \textrm{wound}\ \textrm{area}\right)}{\textrm{first}\ \textrm{day}\ \textrm{wound}\ \textrm{area}}\times 100$$

After the treatment period determined as defined in the experimental design, the animals were sacrificed under general anesthesia on the 10th day. Then, samples were taken from the wound area of the animals of all groups and histopathological analyses were performed.

### Histopathological Analyses

At the end of the investigation, the wound region samples were collected and fixed in 10% neutral formalin during the necropsy for histological and immunohistochemical examinations. To prevent shrinkage at the defect site, the skin samples were secured on a flat surface, and formaldehyde was poured over the area containing the defect. After waiting for 5 min to harden, it was placed in a formalin solution. After a 48-h fixation period, skin samples underwent standard processing through an automated tissue processor (ASP300S; Leica, Wetzlar, Germany). Once embedded in paraffin wax, tissues were sectioned into 5-μm-thick slices using a rotary microtome (Leica RM2155; Leica, Wetzlar, Germany). The histopathological analysis employed the hematoxylin and eosin (HE) method [61], and the resulting sections were coverslipped before examination under a light microscope. The remaining serial sections underwent staining with the Picrosirius red method to assess collagen content (ab150681, Abcam, UK), following the manufacturer’s guidelines. Subsequent examination was carried out under a light microscope. Microphotography and morphometric analyses were conducted using the Database Manual Cell Sens Life Science Imaging Software System (Olympus Corporation, Tokyo, Japan).

### Immunohistochemical Analyses

Chosen sections were subjected to immunostaining for collagen 4 (Anti-Collagen IV antibody, ab6586, Abcam, UK) and cytokeratin (Anti-pan Cytokeratin antibody [C-11], ab7753, Abcam, UK) employing the streptavidin-biotin technique. The primary antibodies were used at a 1/100 dilution, and sections were incubated with them for 60 min. Immunohistochemistry was conducted using a biotinylated secondary antibody and streptavidin–alkaline phosphatase conjugate. The UltraVision Detection System Anti-Polyvalenti HRP kit (TP-060-HL, Thermo Shandon Limited, Cheshire, UK) served as the secondary antibody. Antigens were visualized using diaminobenzidine (DAB) as the chromogen. Omission of the primary antiserum step served as the negative control. All assessments were performed on blinded samples.

All the slides were analyzed for immunopositivity, and semi-quantitative analyses were carried out. Samples were scored from 0 to 3 according to the intensity of staining (0, absence of staining; 1, slight; 2, medium; 3, marked staining). The results were recorded and statistically analyzed. Immunohistochemical score analyses were conducted utilizing ImageJ version 1.48 (National Institutes of Health, Bethesda MD).

### Statistical Analysis

For statistical analysis, the one-way ANOVA Duncan test from the SPSS-22.00 package program was employed to compare the immunohistochemistry scores of the groups. The significance threshold was set at *P* < 0.05.

## Results

### Green Synthesis of Silver Nanoparticles

**Fig. 1:**
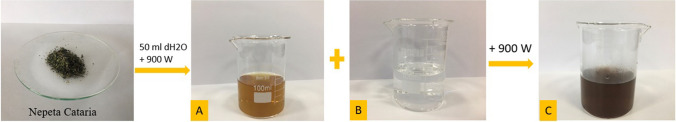
Biosynthesis of silver nanoparticles using *Nepeta cataria* plant extract. **A** *Nepeta cataria* aqueous plant extract. **B** Silver nitrate. **C** Silver nanoparticle (Nc-AgNP)

Silver nanoparticles were synthesized via green synthesis using *Nepeta cataria* aqueous plant extract. The plant extract was yellow (Fig. 1A), and silver nitrate (AgNO_3_) was colorless (Fig. 1B). After adding 5 mM silver nitrate solution to the plant extract and applying heat, the mixture turned dark brown, indicative of silver nanoparticle formation (Fig. 1C).

### Characterization of Silver Nanoparticles

**Fig. 2:**
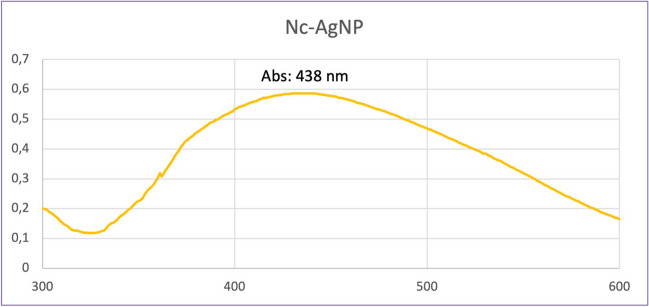
UV-vis spectrum of Nc-AgNPs synthesized using *Nepeta cataria* plant extract

Visual color changes or UV-vis spectrophotometry can be used to monitor the development of AgNPs. Absorption scanning of the synthesized silver nanoparticles was performed with a UV-vis spectrophotometer in the wavelength range of 300–600 nm. Silver nanoparticles (Nc-AgNP) synthesized with *N. cataria* plant extract showed a strong peak at 438 nm (Fig. 2).

**Fig. 3:**
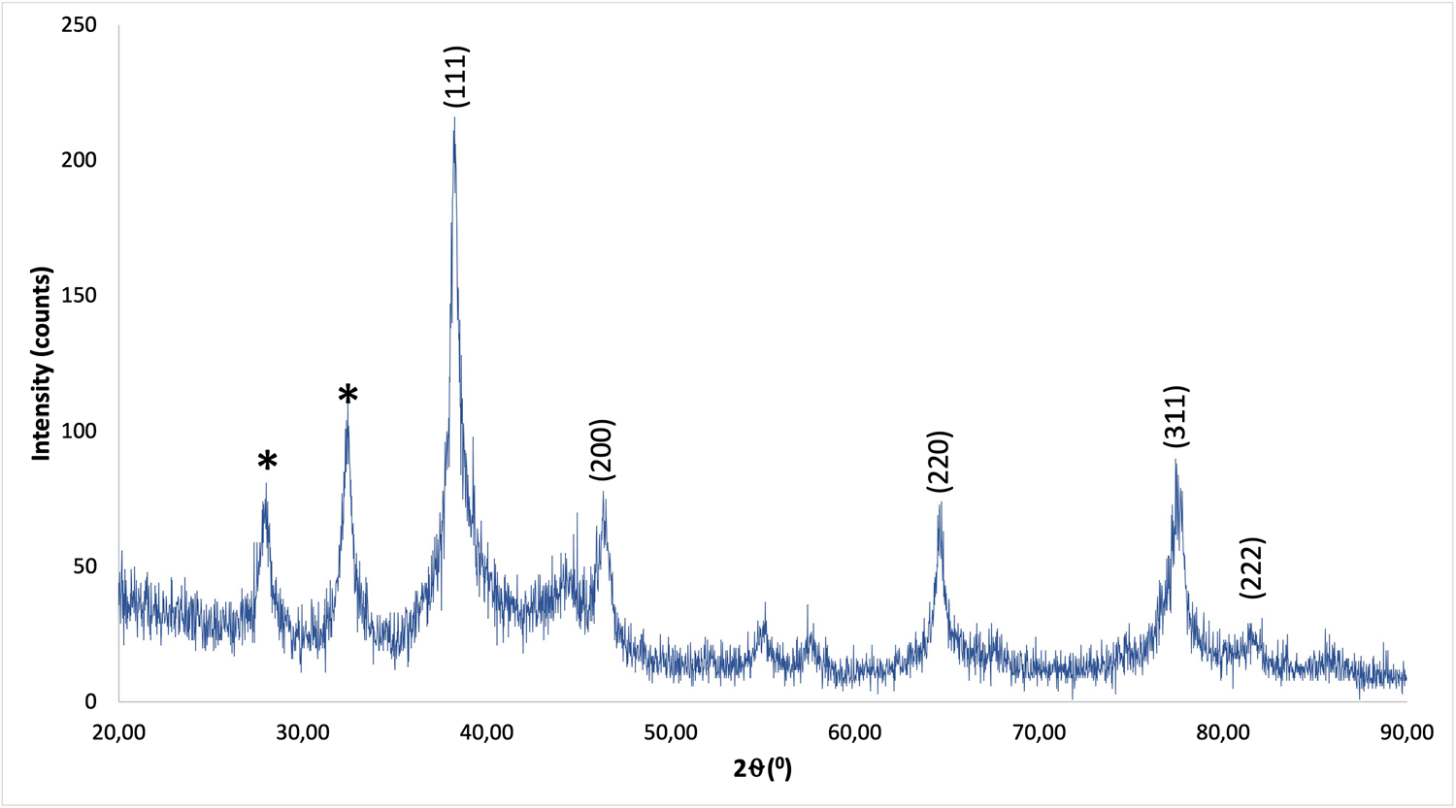
XRD pattern of Nc-AgNPs synthesized using *Nepeta cataria* plant extract

The crystalline structure of Nc-AgNPs was examined via XRD. Five major peaks at 2*θ* values of 38.24°, 46.31°, 64.70°, 77.48°, and 82.11° corresponding to the (111), (200), (220), (311), and (222) planes of silver were observed and compared with the standard powder diffraction card (Joint Committee on Powder Diffraction Standards (JCPDS), silver Card No. 04–0783) (Fig. 3). XRD analysis confirms/shows that the resulting particles (FCC (face-centered cubic)) are silver nanoparticles. At the same time, as seen in the XRD graph, a few unassigned peaks (marked with asterisks) were detected and these peaks were thought to be caused by the organic parts of the plant extract. It is also known that the peak observed at 82.11° originates from the face-centered cubic (FCC) (222) plane. From the XRD pattern, it was seen that the peak belonging to the (111) plane was more intense than the other peak. From this, it was concluded that the silver nanoparticles created in this current synthesis had a crystal structure with an FCC structure. According to XRD analysis, the average crystallite size was calculated as 15.74 nm using the Debre-Scherrer formula (Table 2).

**Table 2:** Crystal size and miller index (Hkl) value of the observed crystal peaks

Peak position 2*θ* (°)	Hkl	Crystalline size (nm)	Average crystalline size (nm)
38.24	111	14.64	15.74
46.31	200	8.68	
64.70	220	16.94	
77.48	311	11.57	
82.11	222	26.85	

**Fig. 4:**
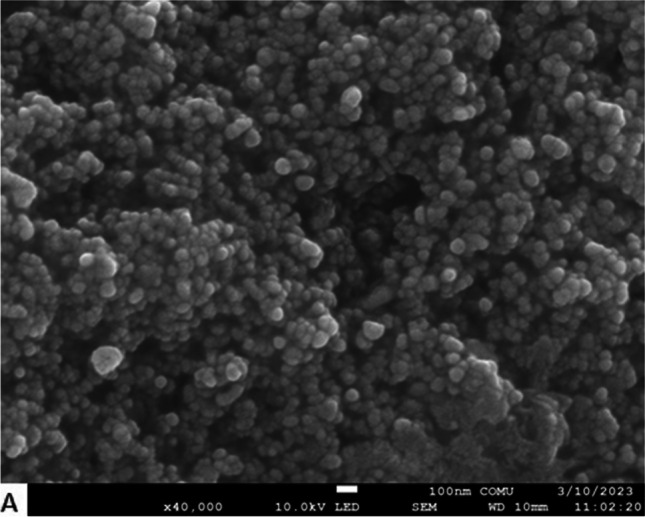
FE-SEM image of Nc-AgNPs synthesized using *Nepeta cataria* plant extract

**Fig. 5:**
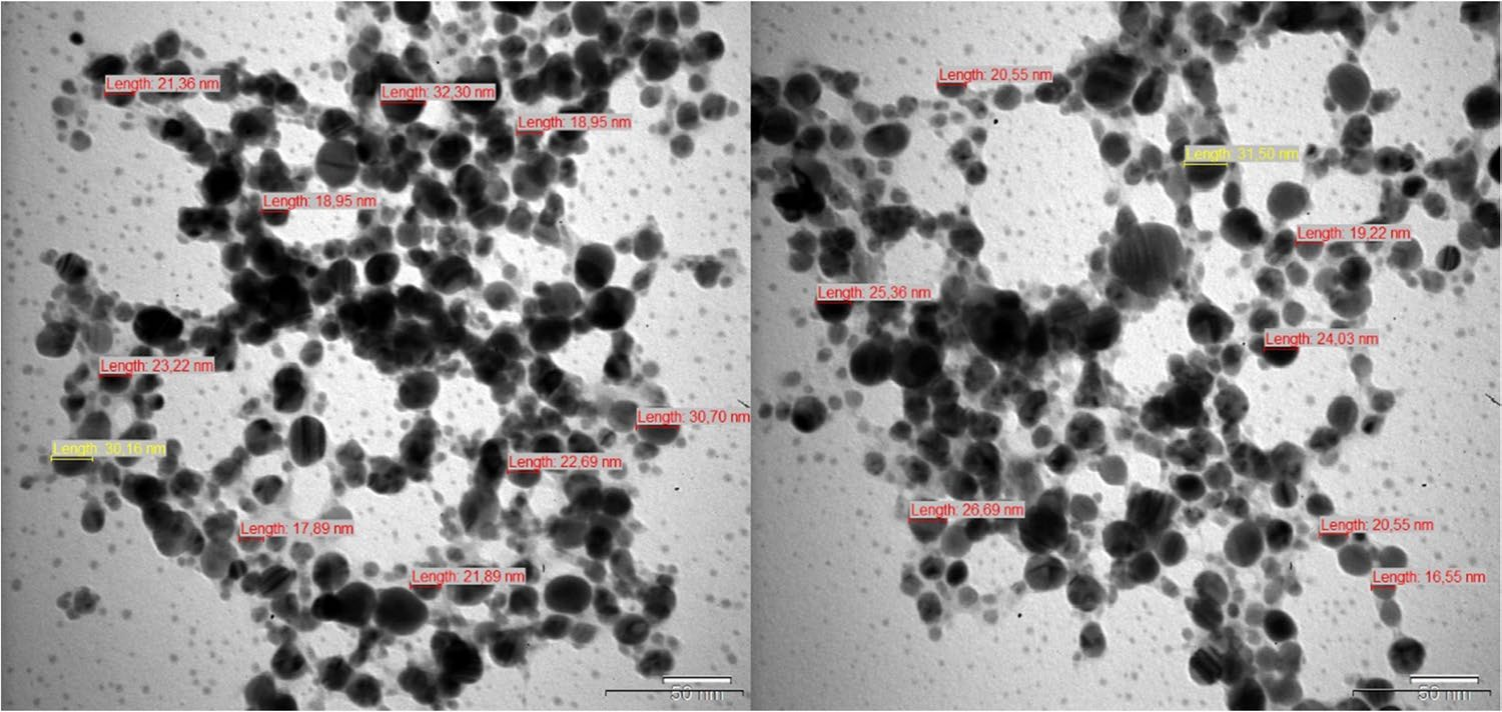
TEM image of Nc-AgNPs synthesized using *Nepeta cataria* plant extract

TEM and FE-SEM were used to determine the morphological properties and size of Nc-AgNPs. FE-SEM images revealed that the biosynthesized Nc-AgNPs had a mainly small size and spherical structure (Fig. 4). At the same time, based on TEM images, the dimensions of Nc-AgNPs were measured as a minimum of 16.55 nm and a maximum of 32.30 nm (Fig. 5).

**Fig. 6:**
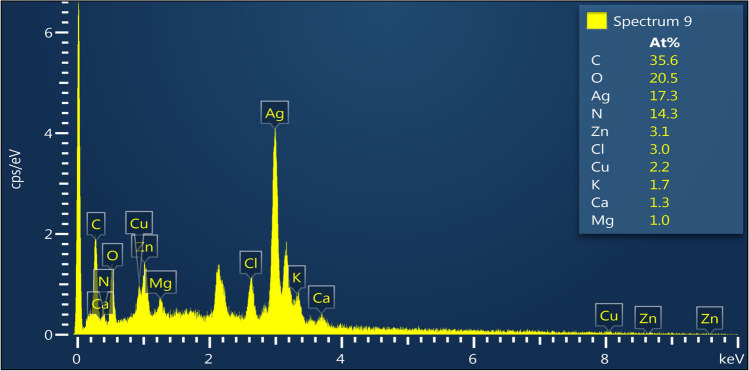
EDX analysis of silver nanoparticles synthesized using *Nepeta cataria* plant extract

EDS analysis was used to evaluate the elemental composition of Nc-AgNPs (Fig. 6). In EDS analysis, the synthesized Nc-AgNPs showed a characteristic absorption peak at 3 keV due to surface plasmon resonance. In addition to Ag (17.3%) in the Nc-AgNP EDS analysis, carbon (C) (35.6%), oxygen (O) (20.5%), nitrogen (N) (14.3%), Zn (3.1%), chlorine (Cl) (3.1%), Cu (2.2%), potassium (K) (1.7%), calcium (Ca) (1.3%), and magnesium (Mg) (1.0%) were determined.

### Antibacterial Activity of Silver Nanoparticles

**Fig. 7:**
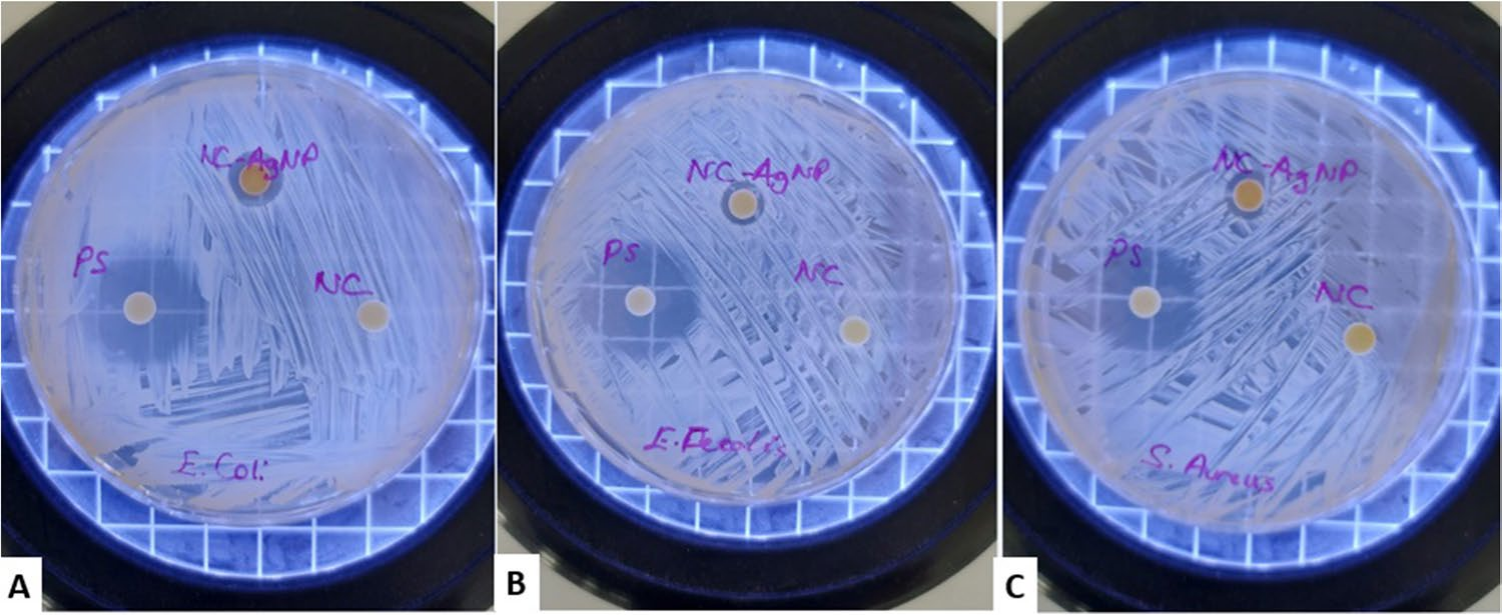
Inhibition zone of green-synthesized Nc-AgNPs against *Escherichia coli* (**A**), *Enterococcus faecalis* (**B**), and *Staphylococcus aureus* (**C**)

Many dangerous bacteria are now resistant to chemical disinfectants and medicines; hence, AgNPs are being employed as antibacterial agents in a variety of settings. Silver is a broad-spectrum antibacterial agent; however, its nanoform may be more beneficial due to its huge surface area, which increases microbial exposure area and duration [62]. The antibacterial efficacy of Nc-AgNPs against three bacterial (*S. aureus*, *E. Fecalis*, and *E.coli*) strains was investigated in this work with the disk diffusion method, and inhibition zones were evaluated. The bacterial activity of Nc-AgNPs was compared with the negative control (sterile water) and the positive control (penicillin/streptomycin). The negative control did not show any antibacterial effect. On the other hand, positive control and Nc-AgNP showed antibacterial effects against gram-negative and gram-positive bacterial strains. *E. coli* showed 12-mm, *E. fecalis* 10-mm, and *S. aureus* 11-mm inhibition zones. Nc-AgNPs showed moderate antibacterial effects compared to the positive control (Fig. 7).

### Antioxidant Activity of Silver Nanoparticles

**Fig. 8:**
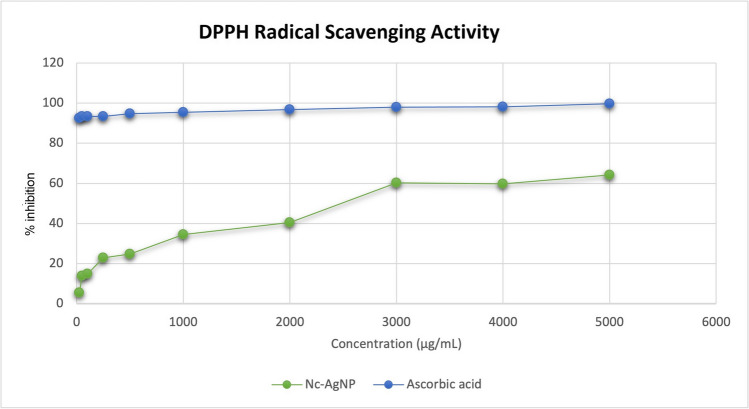
DPPH radical scavenging activity of green-synthesized Nc-AgNPs

Nc-AgNPs were tested against L-ascorbic acid to determine their antioxidant potential. The findings support the antioxidant effect of Nc-AgNPs and L-ascorbic acid. As the concentration of Nc-AgNPs increased, their antioxidant properties also increased. It showed the highest antioxidant activity at 63% at 5000 μg/mL. Nc-AgNPs showed moderate antioxidant activity compared to the positive control, L-ascorbic acid (Fig. 8).

### In Vivo Evaluation of Silver Nanoparticles’ Wound-Healing Efficacy

**Fig. 9:**
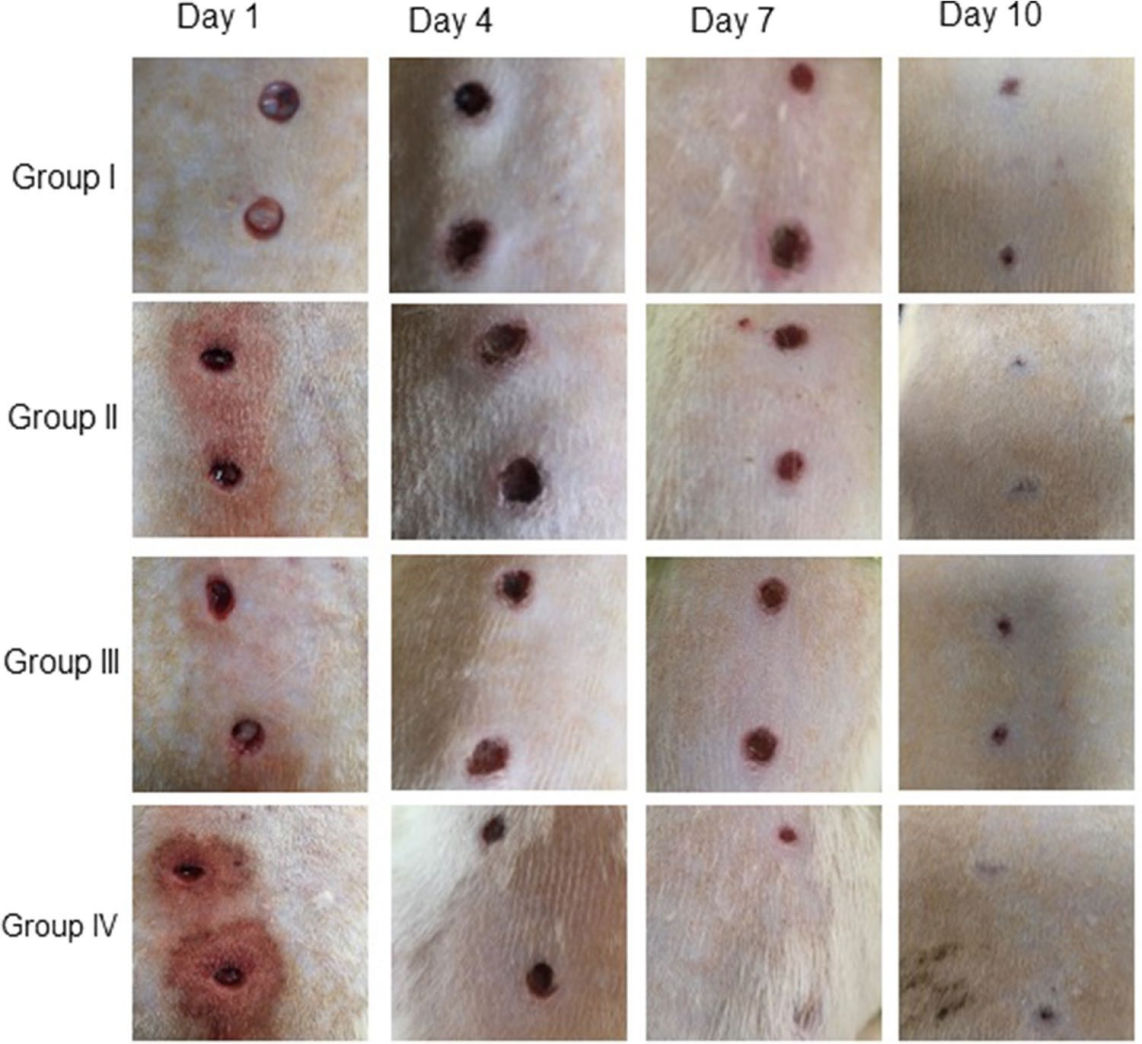
Wound-healing effects of Nc-AgNPs biosynthesized with *Nepeta cataria* extract. Group I (negative control), Group II (1% silver sulfadiazine (Silverdin)), Group III (vaseline), and Group IV (1% Nc-AgNP + Vaseline)

**Fig. 10:**
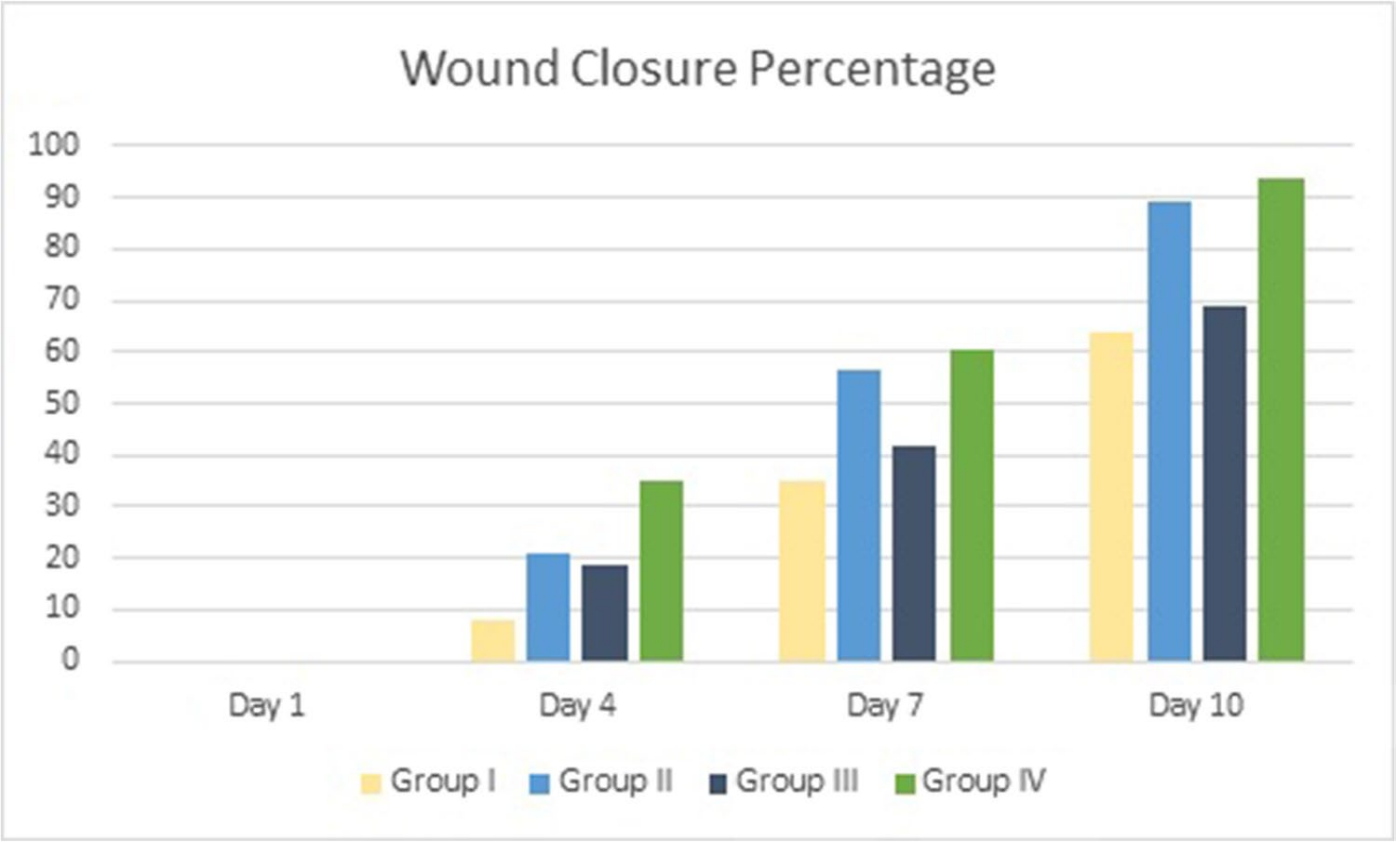
Wound closure percentage of wounds in experimental groups according to days

The wound-healing abilities of Nc-AgNPs were tested using an excision wound model in Wistar albino rats. The wound-healing effectiveness of the developed nanoformulation (Nc-AgNP + Vaseline (1%)) was monitored for 10 days. Following the formation of the wound model, wound area measurements were performed before Nc-AgNP treatment application on days 1, 4, 7, and 10. A rapid closure was observed in all groups starting on the seventh day. The Vaseline + Nc-AgNP treatment group (Group IV) showed a significantly faster recovery compared to the negative control (Group I), vehicle control (Group III), and positive control (Group II) groups. In the measurements made on the 10th day, wound areas decreased significantly in both treatment groups (Group II and Group IV). A visual representation of the wound areas in all experimental groups is provided in Fig. 9. A quantitative evaluation of wound healing is presented in Fig. 10 as the percentage of wound closure.

The wound closure rate in the animal groups was determined to be 94% at the highest level in the group treated with Nc-AgNP + Vaseline after 10 days of treatment. On the other hand, the wound closure percentage was determined to be 89% in the positive control group where Silverdin® was applied, 70% in the vehicle control group where vaseline was applied, and approximately 64% in the negative control group (Fig. 10).

### Histopathological Examination

**Fig. 11:**
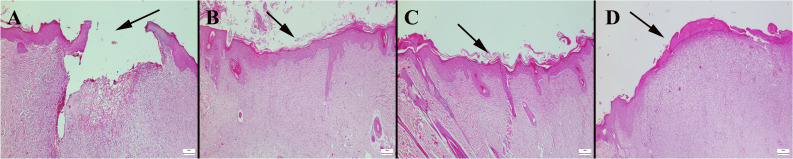
Histopathological appearance of the skin defects between the groups. **A** Unhealed defect area (arrow) in Group I. **B** Totally closed defect area (arrow) and completed epithelization in Group IV. **C** Completed epithelization and fibrous tissue in defect area (arrow) in Group II. **D** Incomplete epithelization but completed fibrous tissue healing in defect area (arrow) in Group III. HE, Scale bars = 200 μm.

In this study, histopathological examination of the defect area in Group I revealed that the wounds of the rats were not fully closed, epithelialization was incomplete, and scab formation was observed at the top of the defect. In Group IV, it was observed that the defect was completely closed and epithelialization was completed. Similarly, in Group II, epithelialization and the development of fibrous connective tissue were completed, and the defects were fully closed. Although healing was not fully formed in Group III, there was a better improvement compared to the control group, with the initiation of epithelialization and the development of connective tissue observed. Thus, it was observed that Nc-AgNPs + Vaseline nanoformulation significantly increased healing similarly to the Silverdin^®^, while Vaseline improved healing compared to Group I but did not result in complete healing (Fig. 11).

**Fig. 12:**
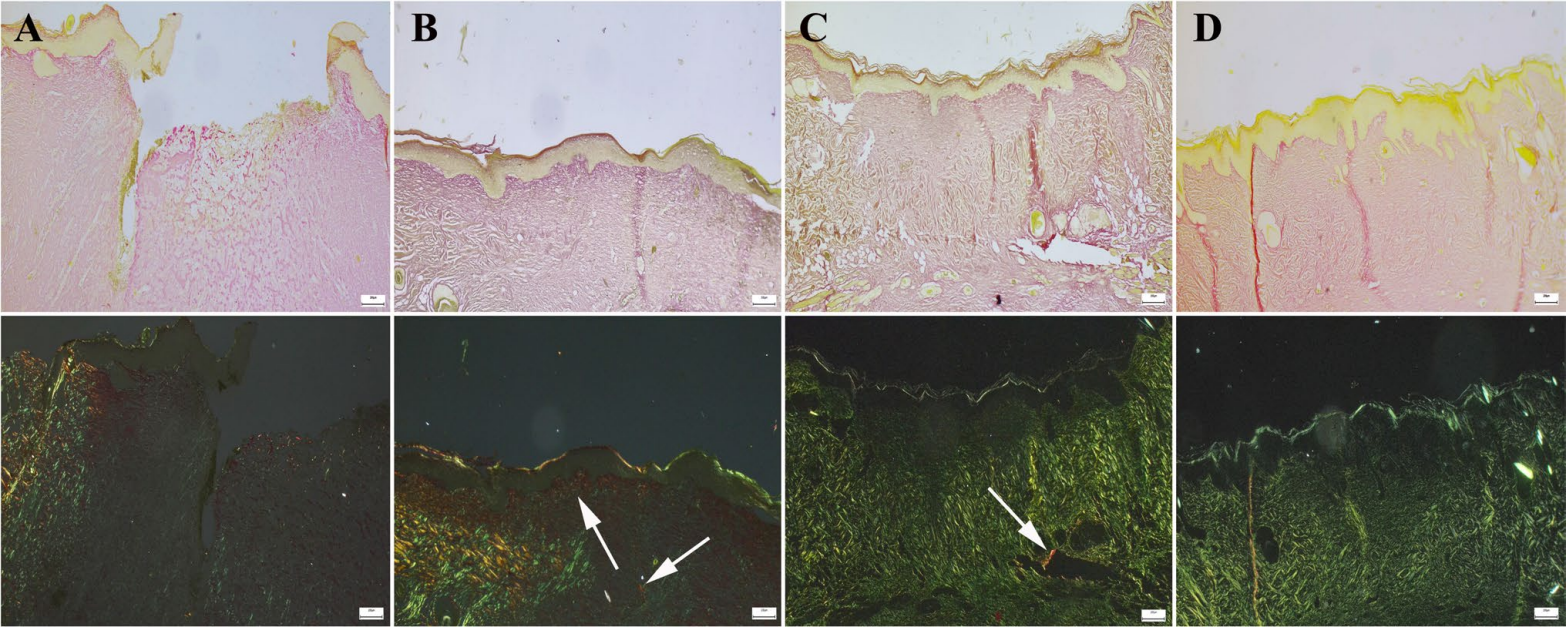
Collagen characteristic of the defect area between the groups (upper row) and same areas under polarized filter (below row). **A** Slight and immature collagen in defect area in Group I. **B** Increased amount of red mature collagen (arrows) in Group IV. **C** Increased mature collagen (arrow) amount in Group II. **D** Marked increase in immature and green Type 3 collagen and slight increase mature red Type 1 collagen in Group III, Picrosirius red method, and scale bars = 200 μm

At Picrosirius red staining, it was observed that in the negative control group, collagen was scarce and immature, predominantly in the form of Type 3 collagen. In Group IV and Group II, an increase in mature Type 1 collagen was noted. In Group III, collagen maturation was found to be better compared to Group I (Fig. 12).

### Immunohistochemical Findings

**Fig. 13:**
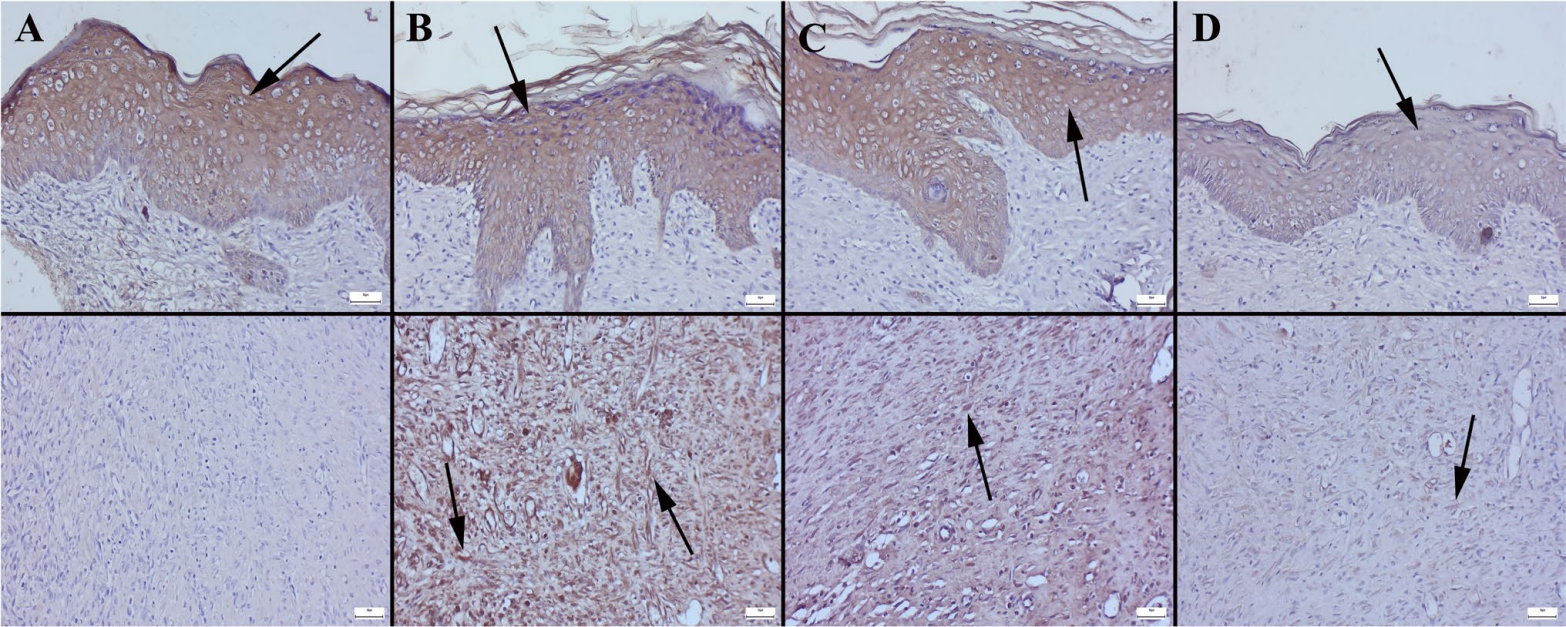
Immunohistochemistry findings (cytokeratin upper row and collagen 4 below row) between the groups. **A** Increased cytokeratin expressions (arrow) epidermal cells only edge of the defect area and slight collagen 4 expression in Group I. **B** Increased cytokeratin and collagen 4 expressions (arrows) in Group IV. **C** Increased cytokeratin and collagen 4 expressions (arrows) in Group II. **D** Moderate cytokeratin and collagen 4 expressions (arrows) in Group III, streptavidin biotin peroxidase method, scale bars = 50 μm

At the examination of cytokeratin-stained preparations evaluating epithelialization, it was observed in Group I that only at the edges of the defect did the initiation of epithelialization occur, with increased expressions in these cells. In Group IV, it was noteworthy that complete epithelialization had taken place, and there were distinct cytokeratin expressions in the epithelial cells. Similarly, in Group II, it was observed that epithelialization was completed and expression increased. In Group III, in some rats, there was evidence of epithelialization, but the expressions were noted to be mild. When collagen expressions were examined according to groups, it was observed that collagen was expressed very slightly in Group I. In Group IV and Group II, a noticeable increase in expressions was evident. In the Group III, moderate increases in expressions were observed (Fig. 13) (Table 3).

**Table 3:** Statistical analysis result of immunohistochemical expression scores between the groups

Groups	Cytokeratin	Collagen 4
Group I	1.50 ± 0.52a	1.60 ± 0.51a
Group II	2.90 ± 0.31b	2.90 ± 0.31b
Group III	1.70 ± 0.48a	1.80 ± 0.63a
Group IV	2.70 ± 0.48b	2.80 ± 0.42b
*P* value	< 0.001	< 0.001

Data expressed mean ± standard deviation (SD). One-way ANOVA Duncan test. The differences between the groups carrying different letters in the same row are statistically significant compared to the CON group, *P* < 0.05

## Discussion

Cuts, burns, diseases (including diabetes), and surgical procedures can all result in wounds. Unfortunately, particularly bacteria can easily contaminate these wounds [63]. Nevertheless, because bacteria in the wound compete with healthy cells for oxygen and nutrients, the presence of endotoxins and metalloproteinases might harm the healing process at any point [27]. A series of molecular, physiological, biochemical, and cellular mechanisms work in tandem to replace damaged structures with new cells and tissues throughout the intricate and dynamic process of wound healing [64]. The field of wound care has advanced significantly since the discovery of antibiotics. Nonetheless, it has been noted that most bacteria eventually start to become resistant to antibiotics [65].

Silver nanoparticles exhibit broad-spectrum antibacterial activities against fungi and bacteria, including antibiotic-resistant strains. Numerous nanoparticles are intrinsic medicinal agents in and of themselves. These days, they are frequently utilized in biomedical applications for managing and healing wounds [66, 67]. Kar et al. assessed the in vivo wound closure percentage of hydrogel-impregnated AgNPs using a rabbit model in their study. They discovered that AgNPs outperformed the positive control (NeuSkin-F) group in wound closure percentage [68].

This study was aimed at producing a safe, biocompatible formulation based on AgNPs for wound healing that is easy to apply and has low toxicity. AgNPs were synthesized using the green synthesis method, which is an environmentally friendly and very economical method, using *Nepeta cataria* plant extract. The *N. cataria* leaf was chosen for the synthesis of AgNPs in this investigation because the leaf extract can decrease the AgNO_3_ solution to AgNPs due to the presence of amines and alkaloids in the leaves, which also act as capping agents [69]. In previous studies, leaves, roots, flowers, fruits, etc. of plants of various species were used for the biosynthesis of AgNPs. In our study, silver nanoparticles were successfully synthesized using *N. cataria* plant extract by the microwave-assisted one-step green synthesis method.

AgNPs absorb radiation in the visible range of the electromagnetic spectrum (400–450 nm) due to the excitation of surface plasmon vibrations, and therefore, AgNPs are observed as brown in many environments [70, 71]. In our study, a brown color transformation of the solution was observed, which was the first finding showing that we successfully produced AgNPs with the green synthesis method. A UV-vis spectrophotometer was also used to aid in the creation of silver nanoparticles. UV-vis spectroscopy is a popular technique for determining the characteristics of nanoparticles. Light wavelengths ranging from 300 to 600 nm are commonly used to examine a wide range of metal NPs with sizes ranging from 2 to 100 nm [54, 72]. Nc-AgNPs synthesized in our study exhibited a strong peak at 438 nm. In the literature, the presence of a peak in the 400–450 nm range is considered an indication that silver nanoparticles have been synthesized [73, 74].

The surface morphology and elemental compositions of Nc-AgNPs were determined by FE-SEM scanning equipped with an EDS detector. In the analyses, it was determined that the synthesized Nc-AgNPs were spherical and smaller than 100 nm in size. The EDS profile of Nc-AgNPs exhibited a strong peak at 3 keV, confirming that the particles were AgNPs [75]. Palani et al. reported a similar result with the peak they identified at 3.3 keV for green-synthesized AgNPs with synthetic melanoidin bacterial extracts [76]. By XRD analysis, the average crystallite size of the nanoparticles was found to be 15.74 nm. Additionally, the structures were determined to be face-centered cubic unit cells. For FCC materials, the high-intensity peak is usually a reflection of the (111) plane peak observed in the sample. The intensity of the peaks reflected the high crystallinity of silver nanoparticles. From the XRD pattern, it was seen that the peak belonging to the (111) plane was more intense than the other peak. From this, it was discovered that the silver nanoparticles produced in this present synthesis were crystalline with the FCC structure. Similar results have been reported in other studies [77–79]. Labulo et al. studies on silver nanoparticles synthesized from *Morinda lucida* plant extract by the green synthesis method were compatible with the findings in this study [54].

Against a wide variety of gram-positive and gram-negative bacteria, AgNPs demonstrated effective potent antibacterial properties. Nevertheless, the precise method by which they exhibit bactericidal or growth-inhibiting activity is still unclear. The available experimental data supports a variety of methods that take into account the physical characteristics of AgNPs, such as their size and surface, which enable them to interact with intracellular components or even pass through membranes and cell walls [56, 80]. In a research by Al-Dbass et al., AgNPs biosynthesized by brown fungi had potent antibacterial activity against bacterial strains of S*. aureus, S. epidermis*, *E. coli*, *S. typhi*, and *P. aeruginosa* [62]. In our study, the antibacterial properties of the synthesized Nc-AgNPs were investigated by the Kirby-Bauer disk diffusion method. As a result, Nc-AgNPs were found to be successful in inhibiting gram-negative and gram-positive (*S. aureus*, *E. faecalis*, and *E.coli*) bacterial strains.

Antioxidants are a type of oxidant antagonist. Antioxidants are naturally occurring or synthesized chemicals that can prevent or postpone cell damage caused by oxidants (reactive oxygen species (ROS), reactive nitrogen species (RNS), free radicals, and other unstable molecules). The ABTS and DPPH tests are two of the most often used methods for measuring antioxidant capability [81]. The DPPH assay was used to determine the *in vitro* antioxidant activity of the synthesized Nc-AgNPs, and it was discovered that the AgNPs had an antioxidant effect. Similarly, antioxidant activities of nanoparticles have been reported in previous studies [82, 83].

In addition, AgNPs’ capacity to heal wounds has been demonstrated to be enhanced by their increased antioxidant activity. Elevated amounts of ROS impede the growth of fibroblasts, and this type of stress damages proteins, lipids, and cell membranes, which delays the healing of wounds [84]. The antioxidant effects of synthesized nanoparticles play a crucial role in promoting wound healing by reducing oxidative stress, enhancing tissue regeneration, modulating inflammation, combating infections, and facilitating the delivery of therapeutic agents to the site of injury.

In our study, the wound-healing properties of Nc-AgNPs were also tested in rats, where an excision wound model was created *in vivo*. Nc-AgNPs produced for use on wound models were integrated into vaseline and turned into a pharmaceutical drug in a silver-based nanoformulation. In Group II (1% silver sulfadiazine cream) and Group IV (Vaseline + Nc-AgNP) (Fig. 9), the wound closure percentage on the 10th day was determined to be 94% and 89%, respectively. These findings revealed that silver-based drugs promote wound healing. These results are in line with previous studies reporting the suitability of silver nanoparticles in dressings, as they also act as a barrier against pathogens, aid wound healing, and facilitate the removal of excess exudate [27].

Wound care is performed every day within the specified period; imaging and millimetric measurements were performed. Except for the negative control group, Silverdin, Vaseline, and Vaseline + Nc-AgNPs were applied topically to the lesions every day, and lesion diameters were measured from the inner edges with a digital caliper (in mm) on days 1, 4, 7, and 10. Wound contraction was calculated as the percentage reduction in wound area as formulated below. Epithelialization time was recorded as the number of days required to ensure that no raw wound was left behind after the wound was opened [60].

AgNPs have been the subject of many studies since the understanding of the effect of silver nanoparticles on wound healing [85, 86]. In our study, the effects of Nc-AgNPs on the excision wound model were examined. Lakkim et al. in a study impregnated AgNPs synthesized from *Catharanthus roseus* and *Azadirachta indica* plants with petroleum jelly and tested their formulation on an excision wound model [48]. The results were similar to our study, and it showed a closure percentage of 93 ± 1% and 86 ± 1%, respectively. Diniz et al. examined the *in vivo* wound-healing effect of silver nanoparticle-based formulations and associated the antibacterial effect caused by silver with its ability to interact with bacterial plasma membranes, proteins, and enzymes involved in vital cellular processes such as the electron transport chain and revealed that silver nanoparticles have different effects on different stages of wound-healing processes [28]. In our study, it was observed that Group IV showed a faster recovery in the first 7 days compared to other groups. This effect may be associated with the first two stages of wound closure, the homeostasis and inflammation phases. Studies are reporting that silver nanoparticles are dangerous for cells, inhibit cell growth and proliferation, and trigger cell death, depending on concentrations and exposure time [66]. In addition to these results, toxicity studies have shown that Nc-AgNPs support wound healing. By activating, migrating, and proliferating epidermal stem cells, a functional layer of keratinocytes is restored, a process known as epithelialization, which is important to wound healing [26]. Silver’s capacity to interact with bacterial plasma membranes, proteins, and enzymes involved in essential cellular functions, like the electron transport chain, has been linked to its antibacterial action [28]. In this study, the groups treated with silver-containing Silverdin® and the Nc-AgNP + vaseline formulation we synthesized showed better recovery than the other groups. This is consistent with other studies showing that silver supports wound healing and epithelialization repair [25]. In his study, he impregnated biologically synthesized silver nanoparticles into the hydrogel and investigated the wound-healing effect of AgNPs by creating an excision wound model on rats. Skin samples from the 0.1% AgNP hydrogel-treated group showed a well-stratified epidermis, complete restoration of epidermal layers, and complete basal, spinosum, and granular layers [25]. An increase in mature Type 1 collagen was seen in Groups IV and II. The collagen fiber is particularly important due to its orientation and organization, which play a vital role in the remodeling phase and the resulting final scar appearance following wound closure [87]. In this study, the increase in Type 1 collagen in the Nc-AgNP + vaseline treatment group shows that AgNPs accelerate and support collagen fiber regulation and wound healing.

## Conclusions

Our study showed that using plant extract in the synthesis process for the preparation of silver nanoparticles is a good option as it is a low-cost, environmentally friendly, and easy method. Silver nanoparticles with an average size of 15.75 nm were successfully synthesized with *Nepeta cataria* (catnip) plant extract. A brown color was obtained, which is indicative of the transition of silver from Ag^+^ to Ag^0^. UV-vis spectrophotometry, FE-SEM, TEM, EDS, and XRD techniques were used to characterize the biosynthesized silver nanoparticles, and the results proved the formation of silver nanoparticles. Studies have shown that Nc-AgNPs, which have antioxidant properties, also have inhibitory properties against gram-negative *Escherichia coli*, gram-positive *Enterococcus faecalis*, and *Staphylococcus aureus* bacteria. Furthermore, in vivo experiments on Wistar albino male rats revealed that Nc-AgNPs significantly enhanced the wound-healing process. By day 10, a remarkable wound closure percentage of 94% was observed, indicating the efficacy of the nanoparticles in promoting rapid tissue regeneration. Histopathologic analyses provided additional evidence of the therapeutic benefits of Nc-AgNPs, showing not only effective wound closure but also robust collagen formation, which is crucial for restoring the structural integrity and strength of the healed tissue. The enhanced wound healing can be attributed to the potent antibacterial properties of Nc-AgNPs, which help in reducing the microbial load at the wound site, thereby preventing infections that could otherwise delay the healing process. Additionally, the antioxidant properties of these nanoparticles likely play a significant role in mitigating oxidative stress, which is known to impede healing. The presence of antioxidants supports the regeneration of healthy tissue by neutralizing free radicals and promoting a more favorable environment for cell proliferation and tissue repair. These findings collectively suggest that silver nanoparticles synthesized using *Nepeta cataria* plant extract hold substantial promise for applications in wound healing. Their ability to effectively combat bacterial infections and support tissue regeneration makes Nc-AgNPs a versatile and powerful tool in the field of regenerative medicine. Consequently, Nc-AgNPs represent a promising candidate for future therapeutic applications aimed at supporting and accelerating the wound-healing process, offering a natural and efficient alternative to conventional treatments.

## Data Availability

No datasets were generated or analysed during the current study.
